# The Relationship between Spinal Stenosis and Neurological Outcome in Traumatic Cervical Spine Injury: An Analysis using Pavlov's Ratio, Spinal Cord Area, and Spinal Canal Area

**DOI:** 10.4055/cios.2009.1.1.11

**Published:** 2009-02-06

**Authors:** Kyung-Jin Song, Byung-Wan Choi, Sul-Jun Kim, Gyu-Hyung Kim, Young-Shin Kim, Ji-Hun Song

**Affiliations:** Department of Orthopedic Surgery, College of Medicine, Research Institute of Clinical Medicine, Chonbuk National University, Jeonju, Korea.; * Department of Orthopedic Surgery, Gwangju Veterans Hospital, Gwangju, Korea.

**Keywords:** Spinal stenosis, Traumatic cervical spine injury, Pavlov's ratio, Spinal cord area, Spinal canal area

## Abstract

**Background:**

This study examined the relationship between four radiological parameters (Pavlov's ratio, sagittal diameter, spinal cord area, and spinal canal area) in patients with a traumatic cervical spine injury, as well as the correlation between these parameters and the neurological outcome.

**Methods:**

A total of 212 cervical spinal levels in 53 patients with a distractive-extension injury were examined. The following four parameters were measured: Pavlov's ratio on the plain lateral radiographs, the sagittal diameter, the spinal cord area, and the spinal canal area on the MRI scans. The Pearson correlation coefficients between the parameters at each level and between the levels of each parameter were evaluated. The correlation between the radiological parameters and the spinal cord injury status classified into four categories, A (complete), B (incomplete), C (radiculopathy), and D (normal) was assessed.

**Results:**

The mean Pavlov's ratio, sagittal diameter, spinal cord area and spinal canal area was 0.84, 12.9 mm, 82.8 mm^2^ and 236.8 mm^2^, respectively. An examination of the correlation between the radiological spinal stenosis and clinical spinal cord injury revealed an increase in the values of the four radiological parameters from cohorts A to D. Pavlov's ratio was the only parameter showing statistically significant correlation with the clinical status (*p* = 0.006).

**Conclusions:**

There was a correlation between the underlying spinal stenosis and the development of neurological impairment after a traumatic cervical spine injury. In addition, it is believed that Pavlov's ratio can be used to help determine and predict the neurological outcome.

The area of the cervical spinal canal is of clinical importance with regard to trauma and degenerative conditions. In addition, some authors reported that the prognosis tends to be worse the smaller the area was.^1^-5) Therefore, some authors recommended prophylactic surgery for paralysis even when the congenital narrowing of the spinal canal was asymptomatic.^1^,5)

The spinal canal area can be evaluated in a variety of ways.^6^-9) One of the most preferred techniques is to measure Pavlov's ratio. Pavlov et al.9) introduced this ratio because it was easy to calculate, useful and unaffected by magnification errors on plain lateral radiographs. The increasing use of CT and MRI has enabled a direct evaluation of the spinal cord area, and some authors reported the clinical outcomes and prognoses of cervical spine injury using these techniques.^4^,^6^,10) According to Okada et al.11), MRI studies of patients with cervical myelopathy reported a correlation between the sagittal diameter of the spinal cord and the severity of the disorder and recovery rate. However, most of these studies were carried out on patients with symptomatic degenerative disease only, not on those with a traumatic injury to the cervical spine. Therefore, they are less helpful in determining the correlation between the underlying spinal stenosis and neurological outcome of trauma. In addition, several studies recently revealed a low correlation between Pavlov's ratio and the spinal cord area measured by MRI,^12^,13) which has created some confusion regarding what radiological parameters can best reflect the development of neurological symptoms.

This study examined the severity of cervical spinal stenosis using Pavlov's ratio on the simple radiographs and the areas of the spinal canal and spinal cord on the MRI scans of patients with a traumatic cervical spine injury. The aim of this study was to elucidate the correlation between the parameters, their association with the neurological status, and the relationship between the vertebral levels using the derived values.

## METHODS

### Materials

Fifty three patients, who were admitted for distractive extension injury between June 2002 and July 2006, were enrolled in this study. Prior to the trauma, none of the patients had neurological symptoms. Patients with spinal cord compression secondary to a traumatic fracture or dislocation were excluded because the aim was to analyze the correlation between the underlying spinal stenosis and the neurological status after trauma. In order to enhance the credibility of this study, an attempt was made to minimize the impact of mechanical compression after trauma on the development of neurological symptoms by including only distractive extension injury. A total of 212 cervical levels from C3 to C6 were examined. There were 44 males and 9 females with a mean age of 54.7 years (range, 21 to 78 years).

### Methods

**Fig. 1:**
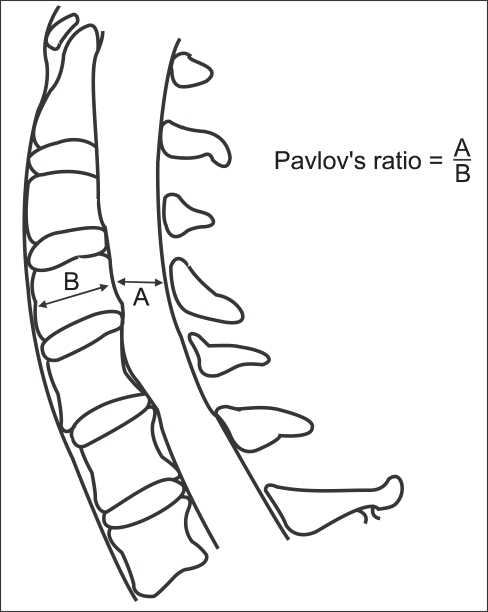
Pavlov's ratio at each level from C3 to C7.

**Fig. 2:**
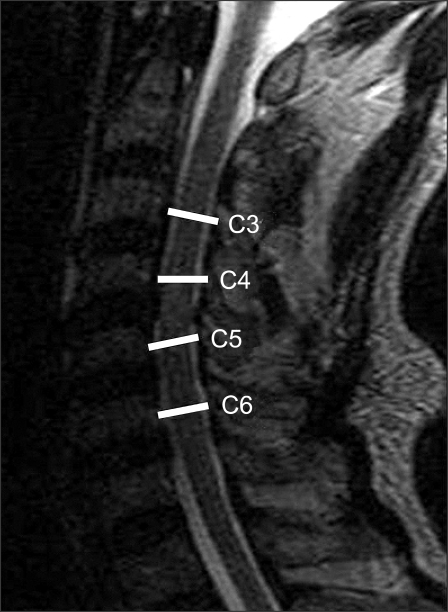
The sagittal diameters of the spinal canal were measured at the midvertebra level on the MR T2 sagittal images from C3 to C7.

**Fig. 3:**
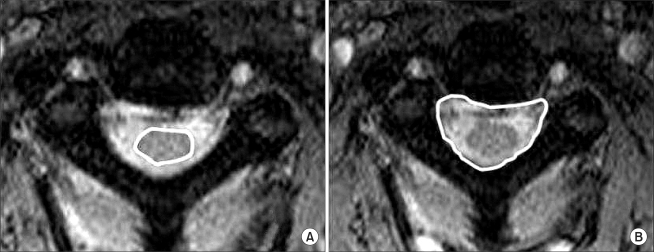
From T2 axial images, (A) the area of the cord, (B) the area of spinal canal area were measured from C3 to C7.

In order to obtain undistorted images, the plain lateral radiographs were taken at a tube distance of 72 inches. The radiation source was 82 kVp 12 MAV X-rays. Pavlov's ratio was calculated using the diameter of the spinal canal and the diameter of the vertebral body at the midpoint from C3 to C6 (Fig. 1). Using these measurements, we confirmed the number of cases with a ratio of ≤ 0.8 indicating radiological stenosis. After the trauma, all patients were examined by MRI (1.5 T Magneton vision, SIEMENS, Elangen, Germany) using a CP spine array coil. The imaging protocol consisted of a sagittal T1-weighted turbo spine-echo sequence (560/13/4/300/256 × 512: TR/TE/excitations/field of view/acquisition matrix), an axial T1-weighted conventional spin-echo sequence (630/14/2/180/144 × 256), an axial gradient-echo sequence (655/27/2/180/144 × 256), and a sagittal T2-weighted turbo spin-echo sequence (3800/115/3/300/256 × 512). The slice thickness for both T1 and T2 scans was 3 mm. On the sagittal T2-weighted images, the diameter of the spinal canal was measured at the midvertebral level from C3 to C7 (Fig. 2). The areas of the spinal cord and spinal canal were calculated from the cross-sectional images (Fig. 3). All the measurements were carried out using a PACS system (m-viewTM, Marotech, Seoul, Korea). Two measurements were taken independently at each level by two observers. The average value provided by each observer was used as a representative value. The reliability of the measured values was examined by evaluating the intraobserver and interobserver agreement using Kappa coefficient.

The average of the following values was obtained: Pavlov's ratio from the simple radiographs, the sagittal diameter of the spinal canal, the area of the spinal cord, and the area of the spinal canal from MRI scans measured at each level from C3 to C6. Pearson correlation analysis and a *p*-value test were used to determine the correlation between the diagnostic tools and measurements. These were also used to examine the relationship between the values derived at each level according to the diagnostic tools. These tests were performed to determine if a value taken at a level can also be indicative of the values taken at the other levels.

The study subjects were divided into 4 cohorts (A, B, C, D) depending on the neurological status in order to determine the association between the severity of spinal stenosis and the post-traumatic neurological deficit in addition to what parameter best reflects the neurological status. According to the severity of the nerve injury, cohort A was defined as those patients with complete paralysis of the motor and sense nerves distal to the trauma site. Cohort B was composed of patients with incomplete paralysis in whom more than half of the major muscles distal to the corresponding level have ≤ grade 3 power. Cohort C consisted of patients with a neurological deficit in whom a decline of the sensor or muscle strength at the corresponding level was present with muscle strength in more than half of the major muscles ≥ grade 4. Cohort D was made up of patients with normal motor and sensor nerves. Seven, 11, 25 and 10 patients fell into cohorts A, B, C and D, respectively. Among the groups, no significant differences were found with regard to gender, age, cause of trauma, and the site of trauma (Table 1). The correlations of Pavlov's ratio, the sagittal diameter of the spinal canal, the areas of the spinal canal and the spinal cord were investigated. For this purpose, statistical analysis was carried out using a one way ANOVA test. A *p* value ≤ 0.05 was considered significant.

## RESULTS

### Values from Plain Radiography and MRI

The measurements taken twice by the two observers were considered reliable. The mean k values for the intraobserver and interobserver reliability were 0.87 and 0.83 respectively. Pavlov's ratio was 0.85 at C5 and C6, and > 0.83 and 0.84 at the other levels. The sagittal diameter on the MRI scans was the largest at C5 with a value of 14.5 mm. With regard to the area of the spinal cord, the highest value was 89.3 mm^2^ at C4 and the lowest value was 79.4 mm^2^ at C3. The 5th and 6th cervical vertebrae had similar values, 84.6 mm^2^ and 84.3 mm^2^ respectively. The area of the spinal canal was largest at C4 and C5 (240.1 mm^2^ and 240.7 mm^2^ respectively), and smallest at C3 (230.9 mm^2^) (Table 2). Of a total of 212 levels, 85 (40%) had a Pavlov's ratio of ≤ 0.8: 16 of the 28 levels (40%) in cohort A; 23 out of 44 levels (52%) in cohort B; 40 out of 100 levels (40%) in cohort C; and 6 out of 40 levels (15%) in cohort D.

### Correlation between Parameters

The area of the spinal cord and spinal canal showed a significant correlation (*p* < 0.05) at all vertebral levels measured. Although there was no significant relationship between Pavlov's ratio and the sagittal diameter on the sagittal plane at C5 (*p* = 0.56), statistical significance was observed at the other levels; C3, C4, and C6 (*p* < 0.01, 0.01, and 0.03 respectively). There was no meaningful correlation with the values of the other parameters except at one or two levels. In particular, the correlations between Pavlov's ratio and the spinal cord area and between the sagittal diameter of the spinal canal and the spinal cord area were poor (Table 3).

### Correlation between Levels

The Pearson correlation coefficient and Pavlov's ratio, which were calculated to determine the correlation between the levels and the obtained values, showed a significant correlation between the levels (*p* < 0.01). In particular, with regard to Pavlov's ratio, the Pearson correlation coefficient itself was close to 1, indicating a high correlation between the levels. This can be interpreted to mean that Pavlov's ratio at a certain level was similar to that at the other levels, suggesting that the condition of a certain vertebral level reflects the status of the other levels. On the other hand, there was no correlation with respect to the sagittal diameter of the spinal canal between C5 and C6 on the MRI scans because the Pearson correlation coefficient was 0 and the *p*-value was 0.96 (Table 4).

### Correlation between the Neurological Status and the Radiological Parameters

All radiological values increased gradually from cohort A through cohorts B and C to cohort D. However, a meaningful correlation was only established with regard to Pavlov's ratio (*p* = 0.006). In particular, a ratio of 0.78 in cohort A (complete paralysis) and 0.96 in cohort D (normal) showed a noticeable difference from 0.83 in cohort B (incomplete paralysis) and 0.82 in cohort C (radioculopathy) (*p* = 0.014). Although a proportional increase was observed in the spinal canal area from cohort A through B and C to cohort D, no statistical significance was observed (*p* = 0.158) (Table 5).

## DISCUSSION

Spinal stenosis has been reported to play a major role in the development of symptoms as well as the prognosis of a variety of disorders including Whiplash injury,4) which is characterized by persistent symptoms and traumatic quadriplegia,14) neuropathy and degenerative cervical spine disorders, such as myelopathy.15) One of the well-established parameters for evaluating cervical spinal stenosis is Pavlov's ratio (spinal canal-vertebral body ratio), which is measured from the plain radiographs. With the development of diagnostic methods, other reliable means for assessing stenosis were introduced, such as the spinal canal area measured from the CT scans and the areas of the spinal cord and spinal canal from the MRI scans.

On the other hand, many studies examined the correlations between the values measured using these diagnostic tools and the clinical symptoms.^7^,8) Lee et al.7) reported that Pavlov's ratio was 0.88 at C3 and C4, and 0.90 at C5 and C6 in ordinary Koreans. In that report, they also identified a low correlation between the spinal canal area and the spinal cord area among the measurements taken, which included the spinal cord area, the dura mater, and the spinal canal area on the MRI scans. In their study, the areas measured from the MRI scans were more closely related to the sagittal diameter of the spinal canal than to Pavlov's ratio from the plain radiographs.

The current study examined patients with a traumatic injury and without neurological deficit prior to the trauma. The mean Pavlov's ratio was 0.85, which is slightly lower than that reported in a previous study, while the areas of the spinal cord and the spinal canal measured from the MRI scans were 82.8 mm^2^ and 236.8 mm^2^ respectively, which is similar to those reported elsewhere.7) In comparison with western studies, the areas of the spinal cord and the spinal canal were similar while Pavlov's ratio was different (ranging from 0.9 and 1.0).^5^,^8^,^13^,15) Pavlov et al.9) analyzed the clinical results based on their invention, Pavlov's ratio. In their study, a ratio of ≥ 1 was defined as normal, and ≤ 0.8 was defined as stenosis with poor clinical outcomes. However, in the index study, 40% of the total levels had a ratio of ≤ 0.8. Moreover, 15% of those in cohort D consisting of patients with good clinical outcomes showed a ratio of ≤ 0.8 ranging from 0.63 to 0.79. This means that the Torg and Pavlov's results should not be applied blindly to Korean patients. In another western study by Kang et al.3) involving traumatic patients, the mean Pavlov's ratio ranged from 0.82 to 0.92 according to the vertebral levels. Therefore, a new threshold is needed with regard to Pavlov's ratio as an indicator of spinal stenosis.

Recent studies reported a poor correlation between Pavlov's ratio on the plain radiographs and the spinal cord area on the other scans.^12^,^13^,16) Such a result suggests that each measurement itself can be regarded as an accurate indicator of spinal stenosis. However, this creates confusion as to what measurements can be reliable and determinant for diagnosing and predicting a clinical condition. With respect to degenerative cervical spine disorders, Penning et al.10) reported that compression of the cord occurs when the cross-sectional area of the cord is < 60 mm^2^ while Houser et al.6) concluded that the shape and degree of flattening of the spinal cord may be an indicator of a neurological deficit and observed spondylosis in 98% of their patients with severe stenosis and a banana-shaped cord. Ono et al.17) reported that the AP compression ratio of < 0.4, which was measured by dividing the sagittal diameter by the transverse diameter of the spinal cord, indicates an abnormal neurological function. Matsuura et al.,8) in their comparative study using CT scans, reported significant differences between normal patients and those with an injury to the spinal cord with regard to the shape and transverse diameter of the spinal canal rather than the area of the spinal cord itself. However, these methods used to determine the shape of the spinal cord and compression ratio can be useful when assessing the severity of a lesion caused by a disc protrusion or osteophytes. However, their use is limited when evaluating traumatic spinal stenosis without severe compression. In this study, the neurological outcome was not affected by the sagittal diameter of the spinal canal and the spinal cord area measured on the MRI images (*p* = 0.539, 0.317) while there was a significant correlation between Pavlov's ratio on the plain radiographs and the development of clinical symptoms (*p* = 0.006). Although the width of the spinal canal area was inversely proportion to the severity of the neurological injury, no statistical significance was found (*p* = 0.158).

With regard to Pavlov's ratio, Torg et al.,5) in their study of football players, concluded that patients with a stable spine should not be banned from participating in sports, even if they had a ratio of ≤ 0.8 and their sensitivity to transient neurapraxia reached 98%. They based their claim on the observation that spinal stenosis did not appear to be a predisposing factor for permanent and severe neurological injury. Hence, they admitted that Pavlov's ratio, albeit useful, could play a limited role in predisposing a patient to complete and incomplete paralyses. Meanwhile, Kang et al.3) reported Pavlov's ratio was quite valuable in terms of its clinical usefulness: patients who had complete spinal cord paralysis and those who had incomplete paralysis were significantly different from patients who sustained a nerve-root injury and those who had no neurological deficit. In addition, there was no remarkable difference between the patients who had sustained a nerve-root injury and those who had no neurological deficit. In the present study, the ratios in cohorts A and D were significantly different from those in cohorts B and C (*p* = 0.014). However, the difference between the latter two groups was minor (*p* = 1.00). Therefore, Pavlov's ratio can help provide a big picture of the patient's future condition, such as complete paralysis, incomplete paralysis and a normal condition. In addition, considering that Pavlov's ratio at a particular level correlated with that at the other levels, it is believed that a value at a certain level can represent the overall condition of the spine.

This study had some of the inherent limitations of any retrospective review. The level of external force at the time of the traumatic injury, which is an important factor in the development of neurological symptoms, could not be evaluated objectively. Accordingly, it cannot be simply concluded that the radiological values had an absolute influence on the clinical outcome. However, the authors of the current study also introduced in a previous study a method in which the level of the external force at the time of trauma and the severity of the damage could be estimated by examining the extent of soft tissue damage using MRI. Therefore, more studies will be needed to analyze the major factors behind the development of the neurological deficit, such as the level of external force, using these indirect methods.

The advantage of this study over previous ones is that the reliability of this study was enhanced by including only those cases with a distractive extension injury and excluding those cases in whom the clinical results of the spinal injury could be affected by mechanical compression caused by bone fragments or dislocations in addition to spinal canal stenosis. In addition, it is believed that the index study can provide useful guidelines for clinical applications. Previous studies on traumatic spinal injury focused on the differences in measurements between the plain radiographs and imaging scans according to the clinical symptoms. In contrast, the correlations between various radiological methods were analyzed to determine which of the radiological parameters could best predict the clinical symptoms.

In a traumatic cervical spinal injury, the level of spinal stenosis appears to affect the neurological outcome. In addition, Pavlov's ratio may be a more effective indicator for determining and predicting the neurological outcome than the areas of the spinal cord and spinal canal.

## Figures and Tables

**Table 1:**
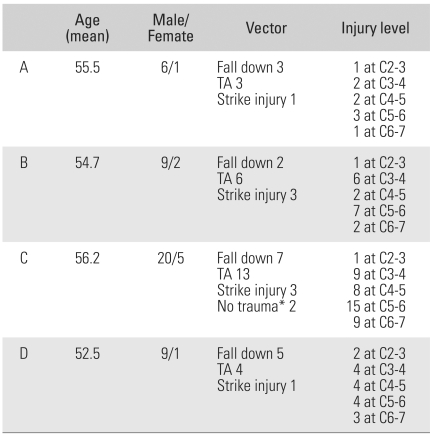
Demographic Data of the Patients

A: Complete injury, B: Incomplete injury, C: Radiculopathy, D: Normal, TA: Traffic accident, ^*^Injury was caused only by excessive extension of the neck by the patient themselves

**Table 2:**
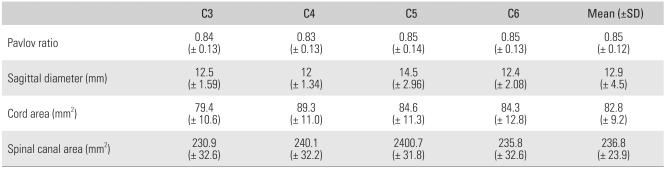
Pavlov Ratio in the Plain X-ray and Sagittal Diameter, Cord Area, Spinal Canal Area in MRI (Mean ± 2 SD)

**Table 3:**
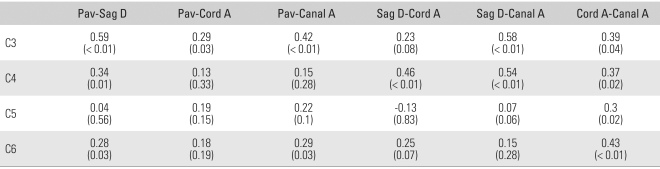
Pearson Correlation Ratio and p-value between the Parameters

Pav: Pavlov ratio, Sag D: Sagittal diameter, Cord A: Cord area, Canal A: Spinal canal area

**Table 4:**
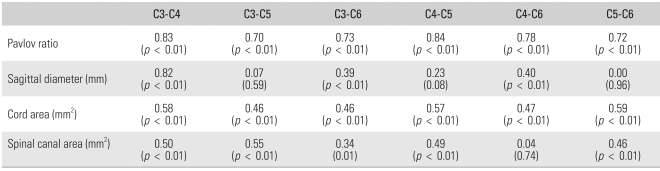
Pearson Correlation Ratio and *p*-value between the Level at Each Parameter

**Table 5:**
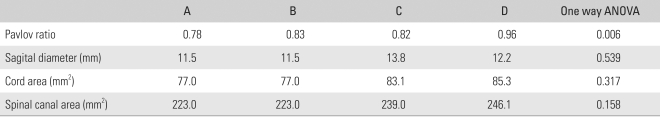
Correlation between the Parameter and Neurologic Status

A: Complete injury, B: Incomplete injury, C: Radiculopathy, D: Normal
